# Sodium lactate for fluid resuscitation: the preferred solution for the coming decades?

**DOI:** 10.1186/cc13973

**Published:** 2014-07-07

**Authors:** Carole Ichai, Jean-Christophe Orban, Eric Fontaine

**Affiliations:** 1 Medicosurgical Intensive Care Unit, Saint Roch University Hospital, 5 rue Pierre Dévoluy, 06000 Nice, France; 2 INSERM, U1081, Institute for Research on Cancer and Aging of Nice (IRCAN), ‘Aging and Diabetes’ Team, 06107 Nice, France; 3 CNRS, UMR7284, IRCAN, Nice, France; 4 University of Nice – Sophia Antipolis, 06000 Nice, France; 5 LBFA – INSERM 1055, University Joseph Fourier, 38000 Grenoble, France

## Abstract

In a recent issue of *Critical Care*, 0.5 M sodium lactate infusion for 24 hours was reported to increase cardiac output in patients with acute heart failure. This effect was associated with a concomitant metabolic alkalosis and a negative water balance. Growing data strongly support the role of lactate as a preferential oxidizable substrate to supply energy metabolism leading to improved organ function (heart and brain especially) in ischemic conditions. Due to its sodium/chloride imbalance, this solution prevents hyperchloremic acidosis and limits fluid overload despite the obligatory high sodium load. Sodium lactate solution therefore shows many advantages and appears a very promising means for resuscitation of critically ill patients. Further studies are needed to establish the most appropriate dose and indications for sodium lactate infusion in order to prevent the occurrence of severe hypernatremia and metabolic alkalosis.

## 

In a recent issue of *Critical Care*, Nalos and colleagues evaluated the effect of 0.5 M sodium lactate (SL) solution on cardiac function in patients presenting acute heart failure [1]. The key result was that 24-hour infusion of SL resulted in increased cardiac output, whereas it did not change in the control group receiving Ringer lactate. This difference could be accounted for, in large part, by an augmentation in stroke volume. Further, patients receiving SL developed metabolic alkalosis, hypokalemia and hypernatremia, while their water balance was strongly negative. These findings highlight the complexity of the beneficial effects induced by SL infusion.

First, hyperlactatemia is a good marker of poor outcome in critically ill patients. Tissue hypoxia is responsible for an increased lactate production resulting from glycolysis [2,3]. Proponents of intensive intervention therefore strongly believed that lactate was, to say the least, a useless waste end product, if not a toxic one. However, the cause–effect relationship is not clearly demonstrated. Indeed, growing data support the notion that lactate production is not deleterious, but rather is an adaptative response allowing one to maintain an appropriate energetic metabolism in the case of shock or organ failure [2-4]. Nevertheless, the effect of solutions containing high lactate concentration in these situations is still a matter of debate.

Evidence is now accumulating that lactate is a preferred substrate readily oxidizable in energy crisis conditions [4-6]. In physiological basal situations, the myocardium functions using energy from beta-oxidation of fatty acids. When oxygen availability is limited, myocardial energy metabolism switches from lipid oxidation to carbohydrate oxidation [7]. Previous studies have already shown that lactate infusion was responsible for an improved myocardial function in patients with septic shock or after cardiac surgery [4,8,9]. The current study reproduces these results in patients with acute heart failure [1]. Levy and colleagues demonstrated that lactate deprivation worsened myocardial metabolism and performance in rats with septic shock [4]. This effect is believed to be due to, for the greater part, the preferential lactate metabolism supplying sufficient energy to the myocardium. However, this hypothesis should be confirmed using direct measurements of lactate oxidation in the heart. Similar results have been reported in normal and injured brain, and confirmed the role of lactate as an important source of energy [6,10,11]. Allaman and colleagues described the essential impact of lactate metabolism in the astrocyte–neuron coupling function [12]. In traumatic brain injury, SL infusion was more efficient than mannitol to decrease raised intracranial pressure [11]. In a later study, a systematic SL infusion over 48 hours decreased by 50% the incidence of elevated intracranial pressure episodes [13].

The second point raised by Nalos and colleagues is the induction of metabolic alkalosis by SL infusion [1], which reflects the probable lactate oxidation. According to the Stewart concept, the exogenous lactate enters into the cells (metabolized anion) while exogenous sodium remains in the plasma (nonmetabolized cation). These modifications in turn induce an increase in the strong ion difference and in the plasma bicarbonate concentration [14]. In agreement with other studies, SL infusion induced metabolic alkalosis and hypokalemia and prevented hyperchloremic acidosis. The authors emphasized that metabolic alkalosis could improve cardiac function [1]. However, it is known that metabolic alkalosis decreases coronary artery blood flow and worsens cardiac arrhythmias, hypokalemia and cardiac contractility. On the other hand, prevention of hyperchloremic acidosis by the balanced SL solution could lead to an improvement in organ function [15,16]. The global consequences of such multiple and intricate metabolic modifications on cardiac function remain questioned.

Third, the infusion of a large amount of sodium in patients with acute heart failure represents a clear danger as sodium restriction is the classic treatment for this condition. Surprisingly, the authors showed that the 24-hour and 48-hour water balances were strongly negative in patients receiving SL compared with the control group. This finding is in agreement with previous studies performed in cardiac surgery and traumatic brain-injured patients [9,11]. The mechanism by which SL influences water balance is not clear. A lower required volume of infusion and/or a higher urine output have been previously reported as a consequence of the sodium/chloride imbalance of this solution [9,11]. Unfortunately, these data are lacking in the study by Nalos and colleagues. Independently of the underlying mechanism, the resulting effect provides a substantial advantage in favor of SL infusion because fluid overload is associated with an increased morbi-mortality in various critical conditions, especially cardiac failure [17].

In conclusion, the study by Nalos and colleagues provides additional arguments to strongly consider SL as a valuable means for resuscitation. Thanks to its high lactate and low chloride concentrations, SL offers substantial clinical improvement in organ functions, especially for the heart and brain.

## Abbreviations

SL: sodium lactate.

## Competing interests

The authors declare that they have no competing interests.
